# Rigidez Arterial na Estenose Aórtica

**DOI:** 10.36660/abc.20201234

**Published:** 2021-03-03

**Affiliations:** 1 Universidade Federal do Rio de Janeiro Rio de JaneiroRJ Brasil Universidade Federal do Rio de Janeiro, Rio de Janeiro, RJ – Brasil.

**Keywords:** Valva Aórtica/cirurgia, Estenose da Valva Aórtica/cirurgia, Substituição da Valva Aórtica/métodos, Análise da Onda de Pulso, Pressão Arterial

O artigo “Alterações da Rigidez Arterial em Pacientes com Estenose Aórtica Grave Submetidos à Cirurgia de Troca Valvar” traz informações importantes sobre o comportamento da rigidez arterial antes e após a troca valvar aórtica.^1^

Eles estudaram 150 pacientes com estenose aórtica grave submetidos à troca valvar aórtica com bioprótese. Eles utilizaram um método não invasivo, a velocidade da onda de pressão (PWV, do inglês *>pressure wave velocity*) carótida-femoral, medida através do aparelho *Complior Analyse* para estudar a rigidez arterial. Outros métodos poderiam ter sido utilizados, como medidas do cateter de pressão invasiva, ressonância magnética e sensores de manguito periféricos, mas a PWV é o padrão-ouro.^2^

O envelhecimento, a hipertensão e a doença aterosclerótica contribuem para o aumento da rigidez vascular medida pela PWV e são fatores de confusão. A idade média de seus pacientes era de 72 ± 8 anos, hipertensão estava presente em 83%, dislipidemia em 76%, diabetes em 35% e 24% tinham história de tabagismo.

Houve associação inversa entre rigidez arterial e gradiente ventrículo esquerdo-aórtico nos pacientes pré-operatórios. Após a troca valvar aórtica, houve aumento significativo da rigidez arterial medida pela PWV, sendo 9,0 ± 2,1 m/s no pré-operatório e 9,9 ± 2,2 m/s (± 2,2 meses após a cirurgia) no pós-operatório. Eles postularam que a obstrução a montante pode interferir nas medidas, mascarando os efeitos reais na aorta. Yotti et al.,^3^ demonstraram que após o alívio da obstrução ocorre um aumento da carga vascular, da pressão arterial e da impedância vascular, induzindo a um comportamento vascular que apresenta rigidez.

O estudo apresenta algumas limitações: centro único, retrospectivo, variabilidade quanto ao tempo de medidas pós-operatórias, falta de randomização, pequeno tamanho da amostra e variabilidade dos dados de acordo com o estado do paciente.

Singh et al.,^4^ estudando 174 pacientes por ressonância magnética, demonstraram que em pacientes com estenose aórtica, aqueles com válvula aórtica bicúspide (VAB) apresentam menor rigidez aórtica quando comparados àqueles com valva tricúspide (VAT) apesar do aumento das dimensões aórticas, mas os autores não encontraram essa diferença, embora não tenha havido menção em relação ao número de pacientes com VAB na amostra.

O impacto do estudo da rigidez arterial merece atenção especial, pois modificações na parede arterial levarão ao aumento da rigidez arterial, que pode ser responsável pelo envelhecimento vascular acelerado e hipertensão arterial; além disso, a rigidez arterial foi incorporada à estratificação de risco de lesões subclínicas de órgãos-alvo.^5^ Vlachopoulos et al.,^6^ mostraram que um aumento da PWV de 1 m/s estava associado a um aumento na mortalidade cardiovascular e por todas as causas. Saeed et al.,^7^ estudando 103 pacientes assintomáticos com estenose aórtica moderada a grave, demonstraram que pacientes com PWV elevada estavam associados a maior risco de doença cardiovascular e morte. Outro problema que às vezes está associado à estenose aórtica é a necessidade de substituir a aorta ascendente por um enxerto, ou implantar uma endoprótese na aorta ascendente, descendente ou arco aórtico. de Beaufort et al., em estudo experimental com 20 pacientes com aortas porcinas, demonstraram que a PWV aumentou significativamente após a implantação da endoprótese.^8^

Como os autores ^1^ afirmam em sua conclusão, o estudo da rigidez arterial pode fornecer melhores esclarecimentos sobre a história natural da estenose aórtica e sua associação com a função vascular.
