# Supplementary material for: Rigidez Arterial na Estenose Aórtica
Source: Arq Bras Cardiol. 2021 Mar 3;116(3):483–4. [Article in Portuguese] doi: 10.36660/abc.20201234 (PMC8159550; doi:10.36660/abc.20201234)
Supplement: Translation

# Translation: Arterial Stiffness in Aortic Stenosis

**Affiliations:** 1 Rio de JaneiroRJ Brazil Universidade Federal do Rio de Janeiro, Rio de Janeiro, RJ – Brazil.

**Keywords:** Aortic Valve/surgery, Aortic Valve Stenosis/surgery, Aortic Valve Replacement/methods, Pulse Wave Analysis, Blood Pressure

The paper “Arterial Stiffness Changes in Severe Aortic Stenosis Patients Submitted to Valve Replacement Surgery” has important information on the behavior of arterial stiffness before and after aortic valve replacement.^1^

They studied 150 patients with severe aortic stenosis submitted to aortic valve replacement with bioprosthesis. They used a noninvasive method, carotid-femoral pressure wave velocity (PWV) measured through Complior Analyse, to study arterial stiffness. Other methods could have been used, such as invasive pressure catheter measurements, MRI and peripheral cuff sensors, but PWV is the gold standard.^2^

Aging, hypertension and atherosclerotic disease contributes to the increase in vascular stiffness as measured by PWV and are confounding factors. Their patients’ mean age was 72± 8 years, hypertension was present in 83%, dyslipidemia in 76%, diabetes in 35% and 24% had a history of smoking.

There was an inverse association between arterial stiffness and the left ventricle-aortic gradient in the preoperative patients. After aortic valve replacement, there was a significant increase in arterial stiffness measured by PWV, being 9.0± 2.1 m/s in the preoperative and 9.9±2.2 m/s (±2.2 months after surgery) in the postoperative period. They postulated that the upstream obstruction might interfere with the measurements, masking the real effects on the aorta. Yotti et al.^3^ have shown that after relieving the obstruction there is an increase in the vascular load, arterial pressure and vascular impedance, inducing a vascular behavior that shows stiffness.

The study has some limitations: single center, retrospective, variability regarding the time of postoperative measurements, lack of randomization, small sample size and variability of data according to patient status.

Singh et al.,^4^ studying 174 patients using MRI have demonstrated that in patients with aortic stenosis, those with bicuspid aortic valve (BAV) have lower aortic stiffness when compared to those with tricuspid valves (TAV), despite increased aortic dimensions, but the authors found no such difference, although there was no mention regarding the number of BAV patients in the sample.

The impact of studying arterial stiffness deserves special attention, as modifications in the arterial wall will lead to an increase in the arterial stiffness, which may be responsible for accelerated vascular aging and arterial hypertension; moreover, arterial stiffness have been incorporated into the risk stratification of subclinical target organ lesions.^5^ Vlachopoulos et al.^6^ have shown that an increase of the PWV of 1 m/s was associated with an increase in cardiovascular and all-cause mortality. Saeed et al.,^7^ studying 103 asymptomatic patients with moderate to severe aortic stenosis, have shown that patients with elevated PWV were associated with a higher risk of cardiovascular disease and death.

Another problem that is sometimes associated with aortic stenosis is the need to replace the ascending aorta with a graft, or implant an endograft in the ascending, descending aorta or aortic arch. de Beaufort et al, in an experimental study with 20 patients with porcine aortas, demonstrated that PWV was significatively increased after stent graft deployment.^8^

As the authors^1^ state in their conclusion, the study of arterial stiffness may give better insights into the natural history of aortic stenosis and its association with the vascular function.
